# Splicing the narrative: alternative *TARDBP* splicing and its relation to neurodegeneration in ALS and FTD

**DOI:** 10.1172/JCI199846

**Published:** 2026-03-16

**Authors:** Morgan R. Miller, Megan Dykstra, Sami Barmada

**Affiliations:** 1 Neuroscience Graduate Program, University of Michigan, Ann Arbor, Michigan, USA.; 2 Biogen, Cambridge, Massachusetts, USA.; 3 Department of Neurology, University of Michigan School of Medicine, Ann Arbor, Michigan, USA.

## Abstract

Amyotrophic lateral sclerosis (ALS) and frontotemporal dementia (FTD) are progressive neurodegenerative diseases characterized by the nuclear clearance and cytoplasmic aggregation of transactive response DNA/RNA-binding protein of 43 kDa (TDP43). Alternative splicing of *TARDBP*, the gene encoding TDP43, leads to a surprising diversity of RNA and protein isoforms with unique functions and potential implications for disease pathogenesis. Here, we review the production, properties, and functional consequences of alternative splicing in the development of ALS and FTD, focusing primarily on TDP43 due to its integral connection with the pathogenesis of sporadic as well as familial forms of these diseases. We synthesize current evidence on the biology of alternative *TARDBP* splicing, highlight key questions regarding its role in TDP43 proteinopathies such as ALS and FTD, and touch on the larger phenomenon of alternative splicing and its relationship to disease.

## Introduction

Amyotrophic lateral sclerosis (ALS) is a fatal neurological disease caused by the irreversible degeneration of both upper and lower motor neurons, resulting in muscle weakness, atrophy, and paralysis. The disease is devastating, with an average survival of just 3–5 years following symptom onset (1, 2). Although genetic therapies such as tofersen have shown promise for the subset (~2%) of individuals carrying pathogenic *SOD1* mutations (3–5), effective disease-modifying treatments are lacking for the large majority of people with ALS.

A growing body of evidence supports fundamental connections between ALS and frontotemporal dementia (FTD), a neurodegenerative condition affecting neurons in the frontal and temporal lobes of the brain (6–9). FTD is the second-most common form of dementia in individuals less than 65 years of age, manifesting predominantly with behavioral and language impairments (10–12). No FDA-approved therapies are available for FTD, other than symptomatic treatments that address depression, anxiety, and psychotic behavior. In approximately 15% of people with FTD, symptoms of motor neuron disease (weakness, atrophy) identical to those of ALS are also present, emphasizing the intrinsic overlap between FTD and ALS (8, 13, 14).

The neuropathology associated with FTD, frontotemporal lobar degeneration (FTLD), is heterogeneous. Approximately 5%–10% of FTLD is characterized by the abnormal aggregation of fused in sarcoma (FUS) (15) and other members of the FET (FUS, Ewing’s sarcoma, and TATA-binding protein–associated factor 15) (16, 17) protein family. FTLD featuring accumulation of the microtubule-binding protein tau (FTLD-Tau) is more common, found in approximately 45% of individuals with FTD (18, 19), while mislocalization of the RNA-binding protein TDP43 (transactive response DNA/RNA-binding protein, 43 kDa; FTLD-TDP) is found in approximately 50% of people with FTD. Importantly, TDP43 mislocalization is also the signature pathological event in ALS, occurring in greater than 95% of people with this disorder (9, 20, 21). These observations, together with the fact that mutations in the gene encoding TDP43 (*TARDBP*) can cause familial ALS and FTD, confirm the pivotal contribution of TDP43 to both conditions.

Alternative splicing is a critical posttranscriptional mechanism that allows a single gene to produce multiple mRNA transcripts, thereby greatly expanding the diversity of the transcriptome and proteome. In the nervous system, where transcriptomic complexity is exceptionally high (22, 23), several essential processes depend on alternative splicing, including neuronal differentiation, synaptic function, and adaptive plasticity (24, 25). However, this same complexity renders neurons particularly vulnerable to splicing defects. Disruptions in splicing regulation — whether due to mutations in *cis*-regulatory elements, changes in RNA-binding proteins, or broader changes in RNA metabolism — can shift the balance among different isoforms, producing variants with altered stability, aggregation propensity, or subcellular distribution (26, 27). Below, we focus on how alternative *TARDBP* splicing shapes TDP43 function and its contribution to disease pathogenesis.

## TDP43 pathology

TDP43 is a ubiquitously expressed RNA-binding protein involved in RNA splicing, stability, transport, and translation (28–32). TDP43 is primarily localized within the nucleus, but in FTLD-TDP and greater than 95% of individuals with ALS, TDP43 is mislocalized to the cytosol in neurons and glia of the central nervous system (CNS) (20, 21). Mutations in *TARDBP* (33) as well as other prevalent genes associated with ALS and FTD, such as *C9orf72*, *NEK1*, *OPTN*, and *GRN*, also result in TDP43 deposition, implying convergent disease mechanisms centering on TDP43 (34, 35). Emphasizing the significance of these findings, TDP43 pathology is evident in a growing list of neurodegenerative conditions collectively referred to as TDP43 proteinopathies, a category that includes limbic-predominant age-related TDP43 encephalopathy (LATE) (36), inclusion body myositis (IBM) (37), Alzheimer disease (38), and Perry syndrome (39).

## Alternative *TARDBP* splicing

**Figure 1:**
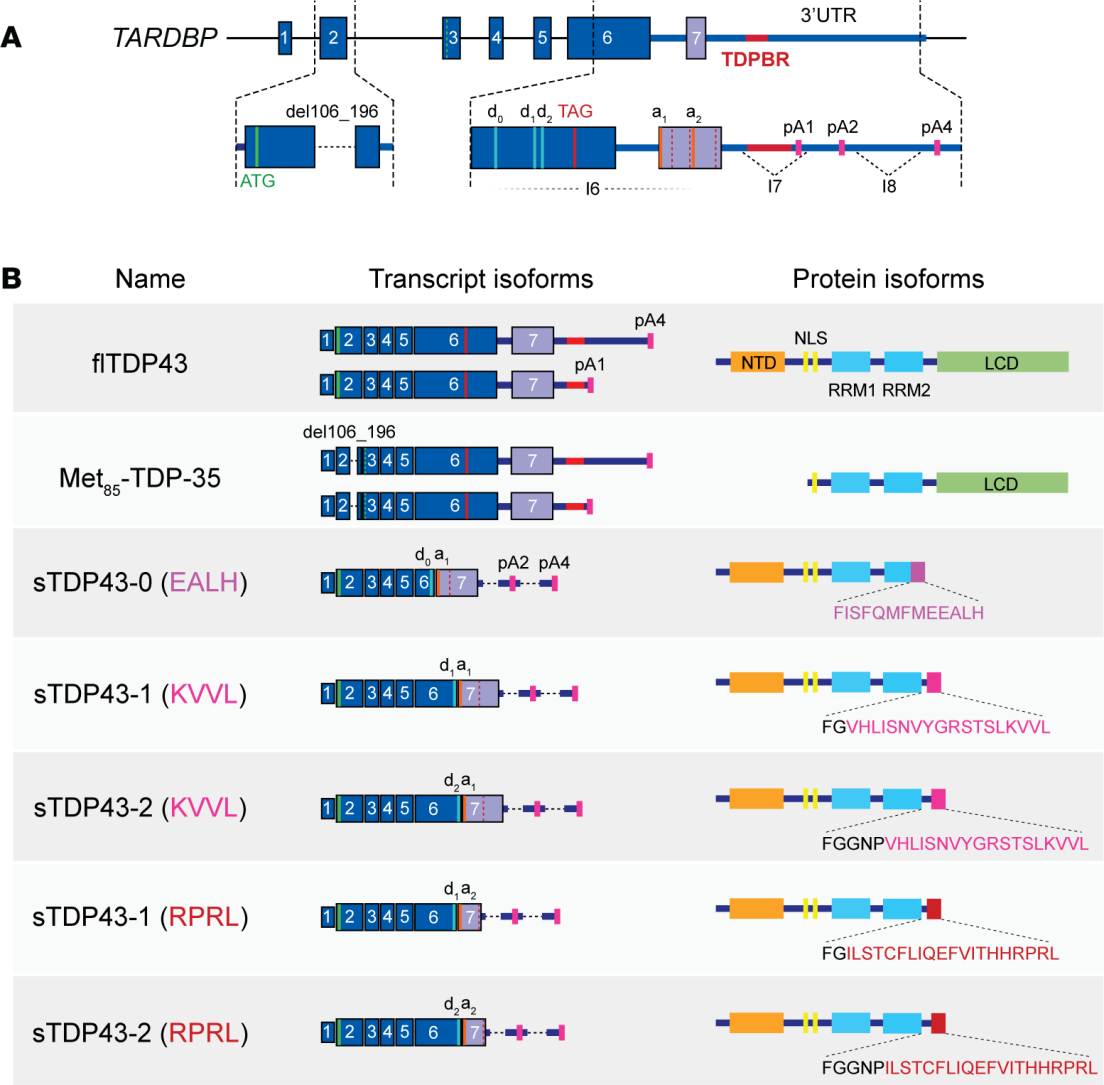
*TARDBP* splicing gives rise to several distinct isoforms. (**A**) Diagram of the *TARDBP* locus, with emphasis on exon 2 and the high splicing density region (HSDR) encompassing exon 6, exon 7, and the proximal 3′ untranslated region (3′UTR). Canonical start (ATG) and stop (TAG) codons are illustrated in exons 2 and 6, respectively. Distinct splice donor (d_0_, d_1_, d_2_) and acceptor (a_1_, a_2_) sites are labeled in the HSDR, as well as polyadenylation (pA) signals, introns 6–8 (I6–I8), and the deletion of nt 96–106 (del96_106) in exon 2. (**B**) Table depicting spliced RNA isoforms and corresponding protein variants for each of the splicing events in **A**. Unique residues encoded by exon 7 are shown in purple (EALH), magenta (KVVL), and red (RPRL), distinguished in each case by the C-terminal 4 aa. NLS, nuclear localization signal; RRM, RNA recognition motif; NTD, N-terminal domain; LCD, low-complexity domain.

Alternative splicing events involving *TARDBP* are concentrated within exon 2, exon 6, and the 3′ untranslated region (3′UTR) (Figure 1). An expressed sequence tag (EST) database search uncovered a splicing event in *TARDBP* exon 2 predicted to eliminate 91 bases from the mature mRNA (40). This deletion results in a frame shift and a premature termination codon (PTC) when translation is initiated at the canonical start codon. However, use of a downstream start codon enables translation of a truncated, 35 kDa protein missing the first 84 amino acids of full-length TDP43 (flTDP43). This isoform, called Met_85_-TDP-35, lacks a portion of the bipartite nuclear localization signal (NLS), resulting in its accumulation within insoluble cytoplasmic aggregates when overexpressed in cultured cells. Antibodies capable of discriminating Met_85_-TDP-35 also label pathological deposits in ALS spinal cord tissue, suggesting that this variant may contribute to TDP43 pathology in vivo. The full extent of *TARDBP* exon 2 splicing is unknown, however, as are the conditions under which this event occurs and the frequency of downstream methionine usage in *TARDBP* translation (40).

Several distinct TDP43 isoforms are generated via splicing within exon 6 and the 3′UTR of *TARDBP* (41–46), a segment dubbed the *TARDBP* high splicing density region (HSDR; Figure 1). Most events occurring within the HSDR result in the excision of a large portion of exon 6, which encodes the C-terminal TDP43 low-complexity domain (LCD). In-frame splicing to cryptic exons within the 3′UTR instead generate three separate C-terminal sequences ranging from 13 to 20 amino acids in length. The predicted protein products from these transcript isoforms all lack the LCD, but retain the flTDP43 N-terminus, NLS, and RNA recognition motifs (RRMs). Evolutionary conservation of the 3′UTR splice acceptors, cryptic exon 7 sequences, and amino acid content for spliced TDP43 (sTDP43) isoforms imply that these variants are critical for neuronal function and/or development (41). Nevertheless, many of the physiological properties of these sTDP43 variants, their distribution, regulation, function, and their potential contribution to neurodegeneration in TDP43 proteinopathies, have yet to be determined.

## *TARDBP* splicing and autoregulation

The splicing events responsible for generating sTDP43 isoforms are triggered by flTDP43 itself through a negative feedback loop known as regulated unproductive splicing and translation (RUST) (47). TDP43 autoregulation is not only efficient in its ability to restore TDP43 protein and mRNA to near-normal levels, but also critical for cellular survival; *TARDBP* knockout is lethal during development, whereas TDP43 overexpression elicits dose-dependent toxicity in multiple cell types and model systems (48–58). In keeping with this, abnormal TDP43 autoregulation is observed in animals harboring a *TARDBP* mutant associated with familial ALS (Q331K), and in postmortem spinal neurons from patients with ALS (59, 60). These observations raise the possibility that TDP43 autoregulation — and by extension, sTDP43 production — may be a fundamental contributor to disease pathogenesis.

The initial steps of autoregulation are triggered by TDP43 recognizing UG-rich sequences present within a defined segment of the *TARDBP* 3′UTR, known as the TDP43-binding region (TDPBR; Figure 1) (61). This results in two potential outcomes: splicing of intron 7 or combined splicing of introns 6 and 7. Removal of intron 7 precludes use of the highly efficient, proximal polyadenylation signal pA1. Subsequent use of the more distal pA2 and pA4 sites leads to nuclear mRNA retention and exosome-mediated transcript degradation (61, 62). In contrast, splicing of both introns 6 and 7 generates sTDP43-encoding transcripts in which a truncated exon 6 is spliced to a “cryptic” exon 7 located within the 3′UTR. These isoforms display a termination codon upstream of the intron 7 splice junction and are thus predicted to be nonsense-mediated RNA decay (NMD) targets. Indeed, blocking NMD through UPF1 knockdown or treatment with cycloheximide, caffeine, or SMG1 inhibitors dramatically increases the abundance of sTDP43 variants (28, 60, 63).

**Figure 2:**
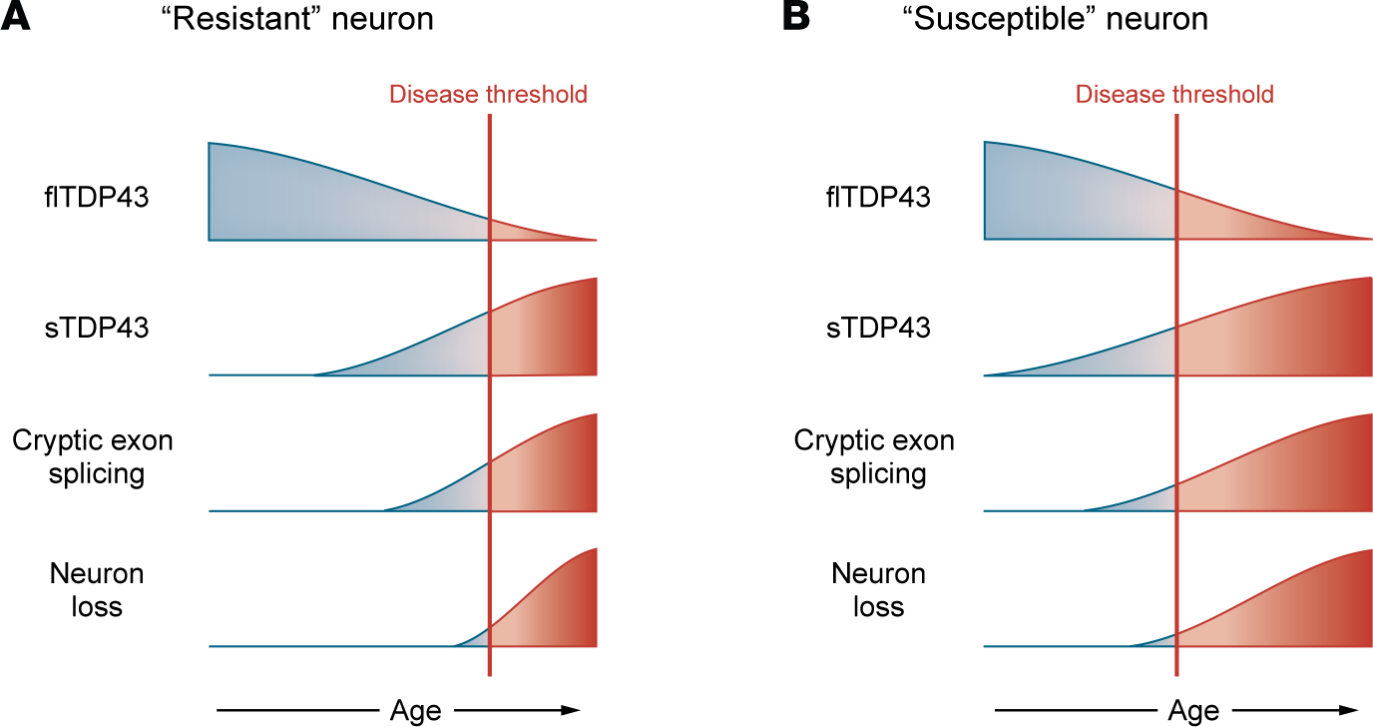
sTDP43 accumulation and TDP43 function are inversely correlated. (**A**) Theoretical timeline depicting the age-related decline in TDP43 function, together with the predicted increase in cryptic splice products. Elevations in sTDP43 may precede reductions in TDP43 function or may compound the physiological decrease in TDP43 that occurs with age, eventually culminating in irreversible neuronal dysfunction and degeneration. (**B**) Susceptible cell types such as motor neurons display relative increases in sTDP43 at baseline, potentially making them more vulnerable to age-related declines in TDP43 function.

Exosome-mediated RNA degradation and NMD are highly effective at regulating sTDP43 variants. Additional mechanisms, including RNA destabilization via miRNAs that recognize motifs within the 3′UTR of sTDP43-encoding transcripts (64), may also contribute to the low abundance of these isoforms in most cell types. Nevertheless, sTDP43 levels display remarkable variability among different species and tissues. Motor neurons, for instance, exhibit 12- to 15-fold higher baseline levels of sTDP43 in comparison with frontal cortex, and the proportion appears to increase with age (41). In keeping with this, although sTDP43 variants are 100- to 1000-fold less abundant than flTDP43 in newly differentiated human induced pluripotent stem cell–derived (iPSC-derived) neurons, they nonetheless represent the majority of *TARDBP* isoforms (>70%) in human spinal neurons isolated by laser capture microdissection from older donors (41). Furthermore, flTDP43 protein levels peak immediately after birth, only to decline slowly with age, and signs of flTDP43 dysfunction (i.e., inclusion of unannotated or “cryptic” exons) are surprisingly common in individuals over the age of 80 (48, 65). Together, these findings indicate that excess TDP43 autoregulation — evidenced by the age-dependent accumulation of sTDP43 — would lead to reduced flTDP43 levels and splicing activity over time (Figure 2). The reasons for age-dependent sTDP43 deposition remain unclear, but possibilities include reduced clearance via the proteosome and/or autophagy (66–68), or reduced fidelity of nuclear import mechanisms (69–71) leading to enhanced cytosolic accumulation of sTDP43.

## sTDP43 protein isoforms

Depending on the specific splice sites utilized, alternative *TARDBP* splicing gives rise to sTDP43 protein isoforms with predicted molecular weights of approximately 25–33 kDa (Figure 1). Given their size, these variants can be confused with C-terminal fragments (CTFs) of TDP43 that are enriched in detergent-insoluble material from FTLD-TDP cortex (21, 72, 73). Appropriate antibodies are therefore critical for characterizing low-MW TDP43 fragments, since sTDP43 variants are selectively detected by N-terminally reactive antibodies, whereas CTFs are only discernible with antibodies raised against C-terminal TDP43 epitopes. In addition, antibodies raised against unique peptides encoded by sequences in exon 7 can help distinguish sTDP43 variants from one another and from flTDP43 proteolytic fragments (41, 42, 45).

Several studies have demonstrated the appearance of 33 kDa proteins detectable using N-terminally–directed TDP43 antibodies; in most cases, these were more abundant upon overexpression of exogenous TDP43, as expected for sTDP43 induction via TDP43 autoregulation (45, 60, 63). The appearance of these low-MW proteins is variable, however, particularly in human cell lines. While the reasons for this are not clear, possibilities include relative scarcity or insolubility of sTDP43 variants, poor immunoreactivity, and/or obscurement of the epitope in question. Notably, this is in direct contrast to mouse tissue, where sTDP43 variants are readily detectable by immunoblotting as well as by mass spectroscopy (42, 43, 63, 74, 75), hinting at species-specific differences in TDP43 autoregulation. The underlying reasons for such discrepancies remain unknown but could include intrinsic differences in the efficiency of RNA clearance mechanisms (i.e., NMD and exosome-mediated RNA decay), TDP43 autoregulation, or protein turnover mechanisms, as described below.

Despite their inconsistent detection by immunoblotting, sTDP43 variants have been repeatedly observed by immunofluorescence and immunohistochemistry in mouse as well as human cell lines and tissue. Most information on sTDP43 distribution arises from studies of the “KVVL” variants (Figure 1). These isoforms differ from one another only by 3 amino acids (residues 281–284), depending on the 5′ splice donor utilized, and are often indistinguishable from one another. Myriad terminologies apply to these variants, including TDP-S6, sTDP43, TDP43-Cspl, and MP18 (Table 1); herein, we will refer to these isoforms simply as sTDP43.

## Phase separation of sTDP43

The LCD located in the flTDP43 C-terminus enables phase separation of TDP43 into liquid-like droplets that effectively concentrate the protein and its substrates within a relatively small area, thereby favoring substrate binding (76, 77). In the absence of the LCD, similarly high local concentrations of protein are incompatible with solubility, leading instead to precipitation and/or the formation of solid aggregates (78). The LCD thus allows for an intermediate biophysical state — a biomolecular condensate — bridging aqueous solubility and frank aggregation, thereby facilitating biological processes through reversible phase transitions.

Notably, many disease-associated RNA-binding proteins prone to aggregation harbor LCDs, and prior studies suggest that the TDP43 LCD is required for protein aggregation and toxicity (79–85). However, more recent investigations emphasize the unique ability of the LCD to prevent irreversible protein aggregation at high local concentrations through phase separation (79). For example, condensation and aggregation of the RNA-binding proteins poly(A)-binding protein 1 (Pab1) and poly(U)-binding protein 1 (Pub1) are paradoxically enhanced by removing the LCD. These studies indicate that RRMs are sufficient for phase separation and aggregation, independent of the LCD (86, 87). sTDP43 variants represent key, naturally occurring examples of this phenomenon; although they lack the entirety of the LCD, sTDP43 isoforms are highly insoluble and prone to aggregation, both in vitro and in cellulo (41, 42, 74).

Although the LCD is instrumental for flTDP43 phase separation, alternative regions of the protein can complement the LCD in promoting condensate formation. Supporting this, TDP43 constructs missing the LCD (ΔLCD) are still capable of undergoing phase separation, albeit at higher concentrations than flTDP43 (78). Biocondensate formation by TDP43(ΔLCD) is dependent on the N-terminal domain (NTD), and swapping the TDP43 NTD to other proteins enables their phase separation (78). In addition, engineered NTD mutations that disrupt flTDP43 oligomerization (i.e., S48E) partially inhibit phase separation (88), suggesting that the NTD is both necessary and sufficient for phase separation. As the sTDP43 NTD is identical to that of flTDP43, it is likely that sTDP43 can undergo phase separation under certain conditions, but this remains to be tested. Moreover, we suspect that posttranslational modifications such as phosphorylation, acetylation, nitrosylation, or cysteine oxidation may interfere with sTDP43 phase separation, as is the case for flTDP43 (89–92).

RNA interactions are crucial for maintaining flTDP43 solubility and phase separation, likely by providing a template for intramolecular interactions (93, 94). Importantly, this is not a simple or unidirectional association; while short RNA substrates that bind only a single molecule of flTDP43 effectively dissolve condensates, longer RNA molecules with a higher motif valency have the opposite effect, enhancing phase separation (95). As with flTDP43, RNA binding may either promote or inhibit sTDP43 condensation, depending on concentration, length, valency, and positioning of binding motifs. Emerging studies capitalized on these observations by designing RNA “decoys” with the ability to dissociate flTDP43 inclusions (96–98). While an RNA decoy comprised of the core 34-nt TDP43 binding region (CLIP34) effectively reversed flTDP43 aggregation in vitro, distinct effects were observed for sTDP43 (99). Unlike TDP43(ΔLCD), which showed no measurable response to CLIP34, sTDP43 fibril formation was paradoxically enhanced upon addition of CLIP34 (99). This is yet another indication of the importance of the sTDP43 C-terminus in determining the biophysical properties of the protein. Additionally, these observations may have substantial implications for the design of RNA decoys, necessitating additional investigations comparing specific RNA sequences and their impact on flTDP43 as well as sTDP43 solubility and localization.

## sTDP43 distribution

Earlier studies suggested the importance of the TDP43 LCD in maintaining protein localization. For example, TDP43(ΔLCD) is diffusely distributed throughout the cell, in contrast to flTDP43, which is concentrated within the nucleus (100). These findings imply that the LCD contributes to nuclear localization, potentially through flTDP43 homo-oligomerization or protein-protein or protein-RNA interactions. Unexpectedly, however, sTDP43 variants do not display such diffuse localization; rather, they are excluded from the nucleus and primarily concentrated within cytosolic granules measuring 100–250 nm in diameter (41, 42, 74, 75). This is true for endogenous sTDP43 as well as epitope-tagged versions of sTDP43 overexpressed in cultured human cell lines, human iPSC-derived neurons, and primary neurons (41, 63). In postmortem brain samples from controls and individuals with ALS, sTDP43 is detectable by immunofluorescence within the cytosol of neurons as well as glia (41, 42). sTDP43 is particularly concentrated within pathological cytosolic TDP43 inclusions, raising the question of whether sTDP43 is a primary driver of flTDP43 mislocalization, or simply a coincidental passenger unrelated to disease pathogenesis.

The differences in behavior between TDP43(ΔLCD) and sTDP43 variants suggest that the unique sTDP43 C-terminus dictates protein localization as well as solubility. Furthermore, despite the presence of a functional NLS, sTDP43 distribution is predominantly cytosolic, indicating dominant mechanisms driven by the C-terminal tail. This region shows little homology to I/L-rich sequences associated with XPO1-dependent nuclear export (101), implying the presence of alternative mechanisms driving sTDP43 aggregation and cytoplasmic deposition. Consistent with this, addition of the sTDP43 “KVVL” C-terminus (Figure 1) to TDP43(ΔLCD) induces the formation of fibrils in vitro (99), correlates with detergent insolubility by immunoblotting, and is sufficient for cytosolic accumulation of an otherwise diffuse EGFP reporter protein (41). Mutating residues within the 18-aa tail eliminates these effects, confirming the importance of this region for sTDP43 aggregation as well as its nuclear exclusion.

RNA binding — one of the most important factors in mediating flTDP43 nuclear retention (102, 103) — also affects sTDP43 localization, but in an opposite manner; mutations that block RNA binding by sTDP43 result in nuclear localization of the typically cytosolic sTDP43 (63). These observations suggest that recognition of cytosolic RNA complexes may help retain sTDP43 within this compartment or shield the NLS from recognition by importins. Alternatively, protein-protein interactions governed by the C-terminal tail of sTDP43 may be pivotal for self-assembly or the formation of large complexes within the cytosol that preclude nuclear import. Future studies will be required to discriminate among these possibilities, and to determine whether distinct C-terminal sTDP43 sequences (i.e., KVVL vs. RPRL vs. EALH; Figure 1) display unique properties.

Biochemical and immunofluorescence studies have repeatedly demonstrated sTDP43’s capacity to physically interact with flTDP43, sequestering flTDP43 within cytosolic inclusions (41, 45, 63). Given the retention of the NTD responsible for dimerization, sTDP43 likely forms a heterodimer with flTDP43, enabling its cytosolic sequestration. This interaction is also associated with nuclear clearance of flTDP43 and a resulting loss of its splicing activity (41, 45, 63). Given the relative abundance of sTDP43 in motor neurons, a cell type that is particularly susceptible to neurodegeneration in ALS, it is possible that sTDP43 helps tip the scale toward flTDP43 mislocalization and dysfunction in these cells. In this way, even a slight increase in sTDP43 levels, together with the age-related reductions in flTDP43 expression, may be sufficient over decades to result in nuclear flTDP43 loss of function and its accumulation into cytosolic deposits characteristic of disease (Figure 2).

The relative scarcity of sTDP43 at the protein level may arise not just from RNA clearance mechanisms, but also from posttranslational protein turnover pathways. Both flTDP43 and sTDP43 are degraded by the ubiquitin-proteasome system (UPS) and autophagy (63, 104–109). However, sTDP43 variants are highly unstable in comparison with flTDP43; while the half-life of flTDP43 ranges from 36–48 hours (104, 105), sTDP43 variants display a half-life of approximately 18 hours (63). Treatment with MG132 (a nonselective cysteine protease inhibitor widely used to suppress proteasomal degradation), or bafilomycin-A1 (a vATPase inhibitor that blocks macroautophagy), effectively stabilizes sTDP43 (63). Moreover, both flTDP43 and sTDP43 are potential substrates for chaperone-mediated autophagy, in which the chaperone HSPA8 delivers proteins directly to lysosomes for degradation (46, 110). For unknown reasons, HSPA8 appears to preferentially recognize sTDP43 over flTDP43, targeting sTDP43 and coaggregating proteins (including flTDP43) for degradation (46).

sTDP43 also displays dramatic species-specific differences in localization and solubility. Endogenous and overexpressed sTDP43 variants are predominantly nuclear in mouse neuroblastoma and microglial cell lines, in contrast with the cytosolic localization of sTDP43 in human cell lines (42, 43). Immunohistochemical staining of endogenous sTDP43 in fixed tissues from mouse brain confirms these differences, and have been repeatedly corroborated using different cell lines, epitope-tagged sTDP43 constructs, and fluorescent sTDP43 variants (41, 45, 63). The reasons for these discrepancies are currently unknown but may be related to the relatively high expression of sTDP43 in rodents, and the distinct nature of TDP43 autoregulation in rodents as compared with humans (45).

## sTDP43 function

The 5′ and 3′ splice junctions involved in the generation of sTDP43, as well as the exon 7 sequences encoding the sTDP43 C-terminus, are highly conserved across evolution, implying an essential, albeit unclear, function for these variants (41). sTDP43 lacks many of the canonical properties of flTDP43 and is ineffective at inducing autoregulation and repressing exon inclusion in the same manner as flTDP43 (41, 63). In keeping with this, both RRM1 and the LCD are required for flTDP43 splicing activity and traditional autoregulation (61). However, sTDP43 can still downregulate the function of flTDP43 via a physical interaction between the proteins, mediated by their respective NTDs. This interaction has two apparent consequences: (a) direct inhibition flTDP43 splicing activity (45, 63), and (b) flTDP43 clearance due to chaperone-mediated autophagy-dependent degradation of the sTDP43-flTDP43 complex (46).

Taken together, these findings suggest that sTDP43 may serve as a critical, short-term regulator of TDP43 activity. The TDP43 negative feedback loop is exceptionally effective at maintaining flTDP43 levels; however, because flTDP43’s half-life is relatively long (~48 hours), it may take several days for flTDP43 levels to return to baseline (53, 63, 105). As described above, neurons and other cell types are exquisitely sensitive to TDP43 levels and may not be able to tolerate such conditions for long. sTDP43 production acutely compensates for this imbalance by rapidly inhibiting flTDP43 splicing activity until protein levels normalize. Furthermore, sTDP43 is quickly cleared (half-life ~18 hours), ensuring balanced regulation of flTDP43 splicing activity over time. In the aging brain, it is possible that elevated sTDP43 production together with inefficient proteolytic cleavage may contribute to abnormal accumulation of sTDP43 and prolonged suppression of flTDP43 function, culminating in TDP43 pathology and missplicing in ALS, FTD, and other TDP43 proteinopathies (Figure 2).

Independent of its effects on flTDP43, two lines of evidence suggest that sTDP43 may also regulate RNA trafficking and metabolism. Like flTDP43, sTDP43 harbors two RRMs that enable RNA binding (63), and overexpressed sTDP43 accumulates within cytosolic stress granules rich in RNA and RNA-binding proteins such as G3BP1, PABC, and eIFη (43, 74, 75). At endogenous levels, however, sTDP43 appears to be only tangentially localized to stress granules and processing (P) bodies, a related type of granule composed of RNA degradation factors (63). There is considerable overlap between these membrane-less organelles and other structures, particularly neuronal activity granules, which are known repositories for cytosolic TDP43 (32, 111). Based on these findings, we suspect that sTDP43 may act as a local transport factor, ferrying RNA substrates among cytosolic membrane-less organelles. Alternatively, sTDP43 could act as a “sheath” for cytosolic mRNA, preventing it from undergoing translation or degradation while in transport to distal compartments (i.e., dendrites, axons, terminals).

## sTDP43 and neuronal activity

**Figure 3:**
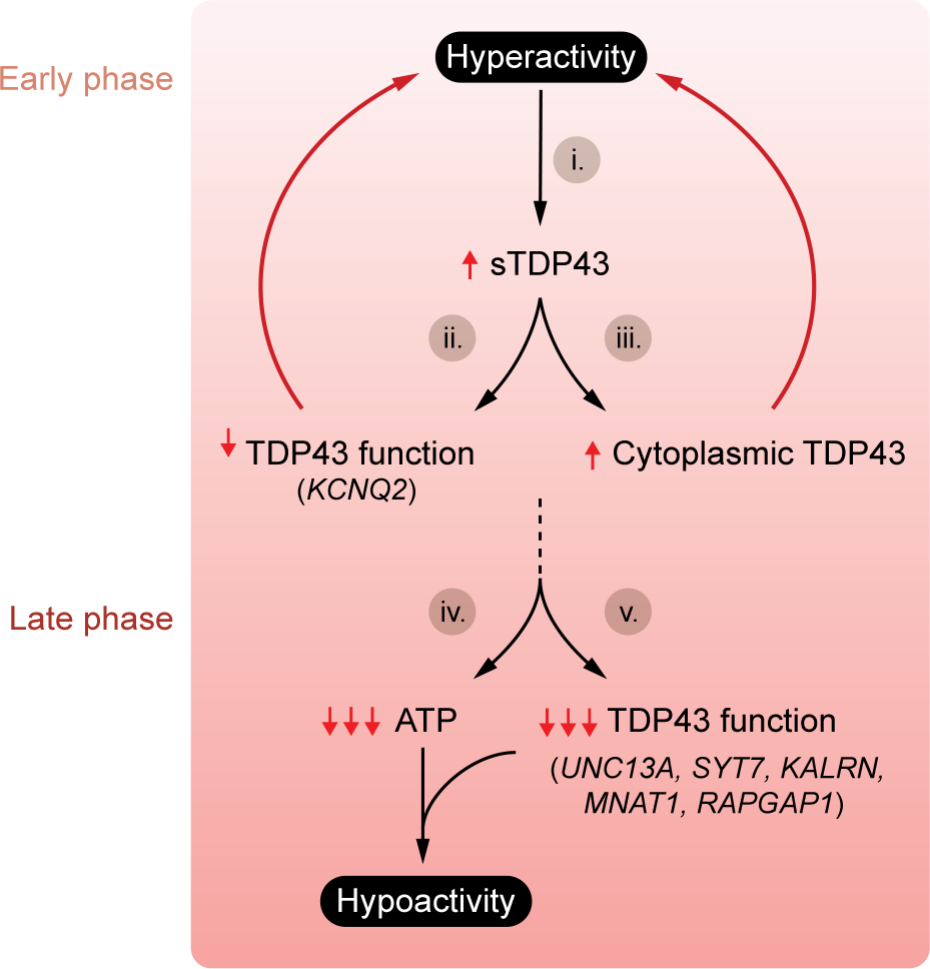
Early neuronal hyperactivity is tied to late-phase hypoactivity in ALS. (i) Neuronal hyperactivity upregulates sTDP43 production, resulting in (ii) reduced flTDP43 splicing activity and the accumulation of cryptic splice products such as *KCNQ2*, reducing membrane potential and contributing to neuronal hyperactivity. sTDP43-induced cytoplasmic aggregation of flTDP43 (iii) also accentuates and promotes hyperactivity, prompting a vicious cycle. Over time, excess firing leads to a reduction in available ATP (iv), ultimately impairing the maintenance of ion gradients required for action potentials, resulting in hypoactivity. Hypoactivity in this model is further aggravated by progressive loss of flTDP43 splicing activity and reductions in key activity-related genes (v).

Neuronal hyperactivity is a prevalent feature of both ALS and FTD, detectable not just in patients via transcranial magnetic stimulation, but also in animal models and isolated human and rodent neurons carrying disease-associated mutations (112–116). In addition, several observations support a fundamental link between neuronal activity and TDP43 pathology. TDP43 is enriched in activity-dependent RNA granules isolated from rodent CNS (117), where it assists in RNA transport and translation in an activity-dependent fashion (118). Moreover, sTDP43 is markedly and selectively upregulated by neuronal hyperactivity (41). Although the mechanisms underlying hyperactivity-induced sTDP43 production remain unclear, this mechanism could explain the nuclear clearance and reduced splicing activity of flTDP43 observed in motor neurons of ALS patients. Overexpression of cytosolic TDP43 lacking an NLS also promotes hyperexcitability in layer V neurons of the motor cortex (115). These observations hint at a feed-forward mechanism in which hyperactivity increases TDP43 levels, initiating autoregulation, sTDP43 production, and flTDP43 sequestration within the cytosol. This, in turn, amplifies hyperexcitability through a vicious cycle (Figure 3).

NMD inhibition may be a critical mechanism contributing to the upregulation of sTDP43 in hyperactive cells. Supporting this, several activity-dependent transcripts (i.e., the activity-related cytoskeleton-associated factor *ARC*) are stabilized upon NMD inhibition (119). Prolonged neuronal activation also triggers the integrated stress response and translational stalling (120); because NMD relies on active translation, hyperactivity could therefore inhibit NMD via its effects on translation. As such, activity-dependent production of sTDP43, arising from the TDP43 autoregulatory pathway, NMD inhibition, or a synergistic combination of both pathways, may promote flTDP43 mislocalization and the loss of flTDP43 splicing activity observed in patients with ALS/FTD.

Prolonged neuronal hyperactivity is inevitably followed by hypoactivity. This transition occurs in aged iPSC-derived neurons carrying disease-associated ALS/FTD mutations, and is mirrored in patients with late-stage ALS/FTD (121–126). One possible explanation for these findings involves the gradual deposition of sTDP43 induced by neuronal hyperactivity (Figure 3). In this model, hyperactivation triggers sTDP43 production and, over time, cytosolic flTDP43 sequestration and loss of flTDP43 splicing activity. Indeed, sTDP43 overexpression disrupts splicing of *STMN2*, a motor neuron–specific flTDP43 substrate, and other transcripts that are regulated by flTDP43 (63). It remains unknown whether sustained sTDP43 expression broadly affects flTDP43 targets that are directly tied to neuronal activity, including *KCNQ2*, *UNC13A*, *SYT7*, and *KALRN* (127–130). If confirmed, these changes could compound deficiencies in available ATP that result from prolonged hyperactivity, culminating in hypoactivity due to a failure to maintain ion gradients required for action potential generation (131). Thus, sTDP43 accumulation may not be a simple consequence of neuronal hyperactivity; rather, it could accentuate hyperactivity while simultaneously facilitating the downstream transition from hyperactivity to hypoactivity, and eventually neuron loss in disease (Figure 3).

## Alternative splicing of related RNA-binding proteins

In addition to *TARDBP*, several other ALS/FTD-linked genes undergo alternative splicing, generating atypical RNA and protein isoforms that have been implicated in disease processes. Many of these alternatively spliced variants exhibit distinct similarity to sTDP43, including production via an autoregulatory loop, relative instability compared with the physiological RNA/protein, sequestration of the physiological isoform, and enrichment in vulnerable motor neurons. Although many of these splice variants are evolutionarily conserved, suggesting canonical roles under normal conditions, age-related changes, impaired proteasomal degradation, or mislocalization of disease-associated proteins can disrupt their regulation, shifting isoform balance toward pathogenic variants that contribute to neurodegeneration.

### SFPQ.

Splicing factor proline- and glutamine-rich (SFPQ) is an RNA-binding protein involved in multiple aspects of RNA metabolism. Similar to TDP43, full-length SFPQ regulates its own expression through a negative feedback loop. In this case, full-length SFPQ induces skipping of exon 10, generating a truncated alternative transcript and protein (altSFPQ) (132). This exon skipping event, which is upregulated in ALS/FTD postmortem samples and disease models, has two primary consequences: first, the altSFPQ transcript harbors a premature termination codon and is therefore a target of NMD. Second, although the majority of altSFPQ transcripts are degraded by NMD, a fraction escapes to be translated into a cytosolically distributed protein. altSFPQ also disrupts the typical nuclear localization of full-length SFPQ, displaying dominant-negative effects akin to those of sTDP43 (132).

### FUS.

Fused in sarcoma (FUS), an RNA-binding protein essential for RNA splicing, transport, stability, and local translation, also regulates the abundance of its own transcripts through an autoregulatory feedback mechanism (133–135). Skipping of *FUS* exon 7 leads to a shift in the reading frame, uncovering a premature termination codon in exon 8, which in turn results in NMD. Exon 7 skipping is reduced in the absence of FUS and enhanced upon FUS overexpression, consistent with autoregulation (135). Elevated nuclear FUS also results in retention of introns 6 and 7, transcript degradation via the nuclear exosome, and reduced FUS translation (134, 136–138). Thus, as with TDP43, FUS autoregulation leverages both exosome-mediated RNA decay and NMD. Notably, both pathways require FUS to act on its own pre-mRNA transcript within the nucleus, and many of the most common of the ALS-associated mutations in FUS affect the C-terminal NLS, resulting in cytosolic FUS mislocalization (139). Under these conditions, rather than restoring homeostasis, the FUS autoregulatory loop could be transformed into a vicious cycle, in which nuclear exclusion of mutant FUS triggers the production of cytosolic protein, which is incapable of negative feedback due to its nuclear exclusion, leading to the production of an ever-increasing amount of FUS.

Embedded within the *FUS* gene is an alternative open reading frame, termed altFUS, encoding a 170-aa protein that is completely distinct from conventional FUS (140). Evolutionary conservation of the altFUS sequence suggests that, like sTDP43, the alternative protein serves an essential function. Through unknown mechanisms, altFUS blocks autophagy, a key pathway for the removal of cytosolic inclusions formed by FUS, TDP43, and other aggregate-prone proteins. Accordingly, altFUS expression facilitates the formation of cytosolic FUS and TDP43 aggregates in cellular models (140).

### hnRNPA1.

Heterogenous nuclear ribonucleoprotein A1 (hnRNPA1) is an RNA-binding protein broadly involved in RNA metabolism, splicing, and transport (141). Alternative splicing gives rise to a variant (hnRNPA1-B) incorporating an extended version of exon 7 that encodes an additional 52 aa (142, 143). The hnRNPA1-B variant is less abundant than the conventional hnRNPA1 isoform in most tissues, but is relatively enriched in the CNS, and becomes more concentrated in ventral motor neurons with age (144, 145). Unlike hnRNPA1, hnRNPA1-B is cytosolic in distribution and highly prone to aggregation when overexpressed in cells (145). Solidifying its connection to disease, hnRNPA1-B splicing is strongly regulated by TDP43. Genetic knockdown of TDP43 using siRNA, mimicking nuclear loss of TDP43 evident in disease, favors hnRNPA1-B production (145). Consistent with this, hnRNPA1-B inclusions are detectable in human ALS spinal neurons exhibiting TDP43 pathology (145). Thus, *hnRNPA1* exon 7B inclusion may serve as a valuable biomarker for TDP43 dysfunction, if not a direct contributor to neurodegeneration in ALS and other TDP43 proteinopathies. 

## Conclusions

Our understanding of TDP43 and its contribution to ALS, FTD, LATE, IBM, and other TDP43 proteinopathies primarily rests on studies of flTDP43 and CTFs. Nevertheless, increasing evidence suggests that alternatively spliced variants of TDP43 assume critical, although incompletely defined, functions in neurons and other cell types. Most of these variants lack the C-terminal LCD and reduce flTDP43 splicing activity through overlapping mechanisms, enhancing the overall efficiency of TDP43 autoregulation. Nevertheless, several key questions remain, including species- and cell-type-specific differences in *TARDBP* splicing that give rise to sTDP43 variants, the factors governing localization, solubility, and abundance of sTDP43 variants, the variety of sTDP43 variants and their functions, and the consequences of sTDP43 deposition in neurons and other cell types.

Perhaps the most important limitation to ongoing investigations of sTDP43 is the lack of high-quality reagents and tools. Antibodies capable of effectively detecting, purifying, and discriminating endogenous sTDP43 variants are critical for accurately defining the localization, levels, interactions, and functions of these isoforms. Circumventing these issues by tagging sTDP43 variants with fluorescent proteins or epitopes creates additional artifacts, since these approaches require protein overexpression that invariably affects sTDP43 solubility, localization, and/or clearance. Moreover, the tags themselves can have effects on sTDP43 subcellular distribution, oligomerization, and its ability to interact with flTDP43. These observations emphasize the critical need for selective and specific sTDP43 antibodies.

Much of our knowledge of TDP43 function stems from *TARDBP*-knockdown or -knockout models. Because of the overlap between flTDP43- and sTDP43-encoding transcripts (Figure 1), however, most knockdown/knockout strategies eliminate not just flTDP43 but also sTDP43 isoforms. Approaches for selectively downregulating or eliminating sTDP43 isoforms without affecting flTDP43, and vice versa, are essential for uncovering distinct functions of flTDP43 as well as each of the individual sTDP43 variants.

Investigations of TDP43 autoregulation and sTDP43 accumulation have been limited by several additional factors. Due to the variability and cell-type specificity of autoregulation, approaches utilizing bulk tissue preparations may be unable to accurately identify *TARDBP* splice variants in vulnerable cell populations such as motor neurons. The instability of alternatively spliced *TARDBP* transcripts also makes them intrinsically difficult to detect (63). More specialized techniques involving subcellular fractionation, or knockdown/inhibition of NMD, exosome-mediated RNA decay, or miRNAs may therefore be more effective at detecting sTDP43 variants in cellular models and postmortem tissues.

Key differences in TDP43 autoregulation between mouse and human tissues also limit the generalization of results from animal models. In general, sTDP43 variants are considerably more abundant in rodent versus human cells, and they display predominantly nuclear localization in rodents rather than the cytosolic distribution observed in humans. The composition of sTDP43 isoforms is also distinct within each species; while sTDP43 variants ending in “RPRL” are more common in humans, rodents almost exclusively express sTDP43 variants ending in “KVVL” (Figure 1) (45). The functional implications of these differences are unclear, as are their potential consequences for age-dependent vulnerability to TDP43 proteinopathies in humans and rodents. Humanized mouse models in which the murine *TARDBP* locus and flanking regions are replaced by human sequences may be exceptionally helpful in navigating these questions (146).

Postmortem tissue, while invaluable for uncovering relevant disease pathophysiology, is plagued by its own complications, including the fundamental restriction to the disease endpoint. This limitation is especially salient for studies of sTDP43, as existing data not only highlight the instability of sTDP43, but also suggest that changes in sTDP43 expression are limited to early stages of disease when neuronal hyperactivity is prevalent. As a result, sTDP43 can be challenging to detect at disease end-stage; rather, postmortem studies have consistently uncovered detergent-insoluble, hyperphosphorylated, CTFs of TDP43 in ALS/FTD cortex and spinal cord (73, 147, 148). Unlike sTDP43, CTFs are generated by posttranslational mechanisms, such as caspase-mediated cleavage of flTDP43 (149, 150). CTFs are also highly stable and prone to aggregation, both in vitro and in vivo, potentially accounting for their relative enrichment in postmortem material. The relationship between sTDP43 and CTFs is unknown, but it is possible that early production of sTDP43 may facilitate late-stage accumulation of CTFs. In this case, excess sTDP43 production — due to a combination of aging, reduced proteolysis, and cell-type-specific splicing — blocks flTDP43 activity and confines it to the cytosol, stimulating autoregulation. As more flTDP43 protein is translated and subsequently trapped within the cytosol, the protein could undergo phosphorylation and/or cleavage, generating CTFs. These fragments may further enhance flTDP43 mislocalization by functional sequestration of essential nucleocytoplasmic transport factors (81). Additional studies are required to test these concepts and define the connections between CTFs and N-terminal TDP43 isoforms such as sTDP43.

Finally, in vitro studies have repeatedly uncovered unique biophysical properties associated with each of the C-terminal sTDP43 sequences, despite their relatively short length (41, 43, 45, 63, 75). Additional experiments contrasting the potential functions, interactions, and expression patterns of each isoform are therefore essential for clarifying their combined influence on neuronal function and survival, in physiological as well as pathophysiological contexts.

## Funding support

This work is the result of NIH funding, in whole or in part, and is subject to the NIH Public Access Policy. Through acceptance of this federal funding, the NIH has been given a right to make the work publicly available in PubMed Central.

- NIH grants R01NS097542 and R37NS113943 (to SJB).
- NIH grant F31NS134123-01 (to MMD).
- NIH grant P30AG072931 (to the University of Michigan Brain Bank and Alzheimer’s Disease Research Center).
- National Science Foundation grant DGE 2241144 (to MRM).
- The family of Angela Dobson and Lyndon Welch.

## Figures and Tables

**Table 1:**
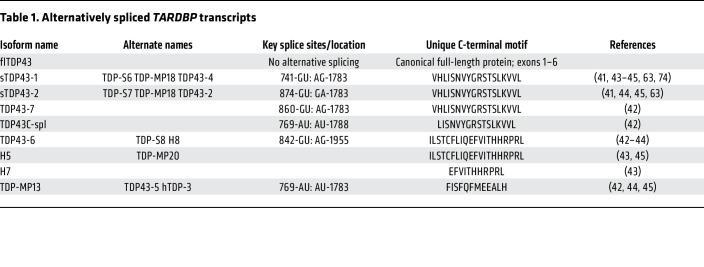
Alternatively spliced *TARDBP* transcripts

## Version 1 03/16/2026

Electronic publication
